# Topical imiquimod yields systemic effects due to unintended oral uptake

**DOI:** 10.1038/srep20134

**Published:** 2016-01-28

**Authors:** Lynda Grine, Sophie Steeland, Sara Van Ryckeghem, Marlies Ballegeer, Stefan Lienenklaus, Siegfried Weiss, Niek N. Sanders, Roosmarijn E. Vandenbroucke, Claude Libert

**Affiliations:** 1 Inflammation Research Center, VIB, Ghent, Belgium; 2 Department of Biomedical Molecular Biology, Ghent University, Ghent, Belgium; 3 Institute for Laboratory Animal Science, Hannover Medical School, Hannover, Germany; 4 Institute for Experimental Infection Research, TWINCORE, Centre for Experimental and Clinical Infection Research, Hannover, Germany; 5 Molecular Immunology, Helmholtz Center for Infection Research, Braunschweig, Germany; 6 Laboratory of Gene Therapy, Faculty of Veterinary Sciences, Ghent University, Ghent, Belgium

## Abstract

Repetitive application of topical imiquimod is used as an experimental model for the induction of psoriasiform skin lesions in mice. The model is characterized by several inflammatory processes, including cytokine production both locally and systemically, cellular infiltration, and splenomegaly. To investigate the production of type I interferons in response to imiquimod-containing Aldara cream, IFNβ-luciferase reporter mice were imaged *in vivo* and *ex vivo*. Type I interferons were found to be produced in the skin, but also in the intestinal system caused by unintended ingestion of imiquimod by the mice. Through the use of Elizabethan collars to prevent ingestion, these effects, including psoriasiform lesions were nearly completely prevented. Our findings reveal that topical treatment with Aldara induces a psoriasiform skin inflammation, but that its mode of action depends on ingestion of the chemical, which leads to systemic responses and affects local inflammation. Therefore, potential ingestion of topical treatments during experimental procedures should be taken into account during assessment of cutaneous inflammatory parameters in skin disease models.

With the growing prevalence of autoimmune diseases, including psoriasis, reliable mouse models have become essential for the discovery and validation of novel drug targets for these diseases and understanding the underlying mechanisms. One expedient model for psoriasis-like inflammation consists of the application of Aldara cream, containing 5% imiquimod (IMQ), on the shaved backs of mice for several consecutive days^1^. IMQ activates the Toll-like receptors 7 and 8 (TLR7/8), and is used to treat genital warts in patients. We and others have shown that besides Tumor Necrosis Factor (TNF), type I interferons (IFNs) are also essential mediators in the Aldara model^2^,^3^,^4^. Although the use of Aldara as a mouse model of psoriasis-like inflammation has increased over time, its mode of action is still incompletely understood. For instance, although it is known that type I IFNs are produced in response to Aldara, it is still debated whether these cytokines are actually involved in the development of the characteristic psoriasiform skin lesions in the model^3^,^5^,^6^. To elucidate the role of type I IFNs in the Aldara model, we aimed to identify the production pattern of type I IFNs. In the current study we report our results in which, surprisingly, we found that the unintentional ingestion of Aldara results in an inflammatory loop in which the intestinal system enhances inflammation in the skin via these type I IFNs.

## Results and Discussion

**Figure 1:**
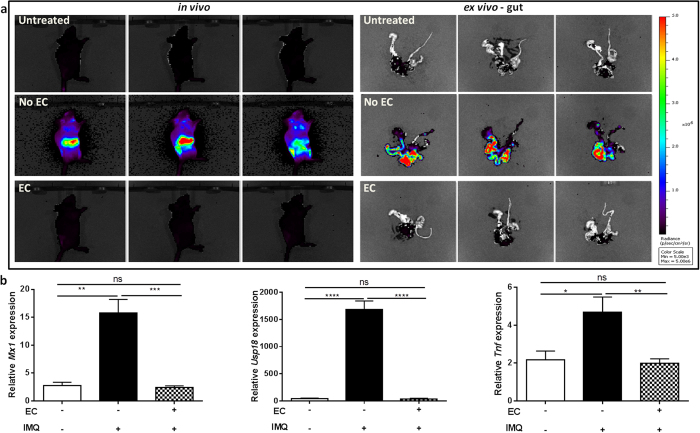
Type I IFNs are induced and activated in the gut after ingestion of topical IMQ, but can be prevented by Elizabethan collars. (**a**) *In vivo* and *ex vivo* imaging of IFNβ-luciferase reporter mice before and 4 h after Aldara (IMQ) treatment. Four hours after Aldara treatment gut was isolated for *ex vivo* imaging. (**b**) Relative expression of ISRE genes *Mx1, Usp18,* and *Tnf* in the gut 4 hours after Aldara (IMQ) treatment. Bars represent mean ± SEM and qPCR data are normalized to the stable reference genes; n = 6 per group *EC: Elizabethan collar; white bar* = *without EC, without IMQ; black bar* = *without EC*, *with IMQ*; *dotted bar* = *with EC, with IMQ*.

In order to study the production pattern of type I IFNs, we treated mice expressing a firefly luciferase gene under the control of the IFNβ promoter (IFNβ-luc)^7^ with Aldara on the skin. Surprisingly, *in vivo* imaging revealed a strong signal in the abdomen of the mice shortly after application (Fig. 1a, left), and *ex vivo* imaging revealed that IFNβ is produced by the gut (Fig. 1a, right). Additionally, a expressions of genes with a typical interferon-stimulated response element (ISRE) such as *Mx1* and *Usp18* (Fig. 1b, left and middle) were detected in the gut. Similarly, *Tnf* was produced as well (Fig. 1b, right). As we noticed that Aldara-treated mice were licking their backs, we investigated whether these changes in gene expression were caused by the ingestion of Aldara and whether ingested Aldara may affect the severity of skin inflammation.

**Figure 2:**
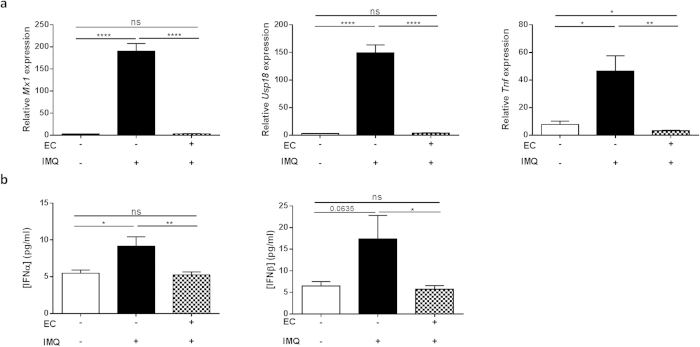
Ingestion of IMQ leads to systemic type I IFN activation, but can be prevented by Elizabethan collars. Mice were treated with Aldara (IMQ) and blood and spleen were isolated 4 hours after application. One group wears ECs, preventing oral uptake of IMQ. (**a**) Relative expression of *Mx1*, *Usp18*, and *TNF* in spleen 4 hours after treatment. (**b**) Using Luminex technology, IFNα (left) and IFNβ (right) were measured in serum 4 hours after treatment. Bars represent mean ± SEM and qPCR data are normalized to the stable reference genes; n = 6 per group *EC: Elizabethan collar; white bar = without EC, without IMQ; black bar = without EC, with IMQ; dotted bar = with EC, with IMQ.*

**Figure 3:**
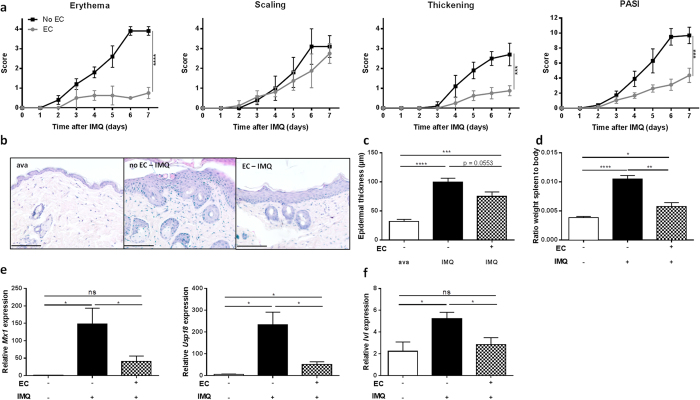
Elizabethan collars prevent full blown IMQ-induced psoriasiform skin inflammation. Mice were treated with Aldara (IMQ) and scored for psoriatic lesions as described previously. One group wore ECs, preventing oral uptake of IMQ. (**a**) Erythema, scaling, thickening and PASI were scored as described previously^1^ (score 0-4). (**b**) Representative skin sections of treated mice taken on day 6 stained with H&E. Scale bars represent 100 μm. (**c**) Epidermal hyperplasia in skin sections taken on day 6, quantified with Volocity. (**d**) Splenomegaly after 6 days, calculated as the ratio of spleen weight to total body weight. (**e**) Relative expression of *Mx1* and *Usp18* in whole skin lysates 4 hours after treatment. (**f**) Relative expression of psoriatic markers genes involucrin (*Ivl*) in whole skin lysates 4 hours after treatment. Bars represent mean ± SEM and qPCR data are normalized to the stable reference genes; n = 5–6 per group *EC: Elizabethan collar; white bar = without EC, without IMQ; black bar/curve = without EC, with IMQ; dotted bar/grey curve = with EC, with IMQ.*

To prevent mice from licking their back skin, a small plastic “Elizabethan collar” (EC) was applied. In contrast to IFNβ-reporter mice lacking such collars, mice wearing ECs displayed no luciferase signal (Fig. 1a, panel below) and did not display an ISRE signature in the gut after Aldara treatment (Fig. 1b, dotted bars). Expression of *Tnf* in the gut was also prevented. Then, we wondered if the systemic production and actions of type I IFNs were affected by oral uptake as well. In mice without ECs, we found a significant upregulation of *Mx1*, *Usp18* and *Tnf* in the spleen and increased IFNα and IFNβ levels in circulation (Fig. 2a,b, black bars). However, these increments were lost in mice wearing ECs (Fig. 2a,b, dotted bars). More importantly, the typical features of Aldara-induced skin inflammation (erythema, scaling and epidermal hyperplasia), represented here as a modified Psoriasis Area Severity Index (PASI) score, were significantly reduced in EC-wearing mice, except scaling (Fig. 3a). We believe the latter is due to the restricted mobility of the mice and the consequence hereof on grooming and barbering. Epidermal hyperplasia was also markedly lower in the EC-group (Fig. 3b,c). Prevention of oral uptake by ECs also affected Aldara-induced splenomegaly as illustrated in Fig. 3d. Analysis of the skin, revealed that induction of the ISRE genes *Mx1* and *Usp18* was also less pronounced in the presence of ECs (Fig. 3e). The expression of the differentiation gene Involucrin (*Ivl*), which is increased during Aldara treatment, was not affected by the Elizabethan collars (Fig. 3f).

**Figure 4:**
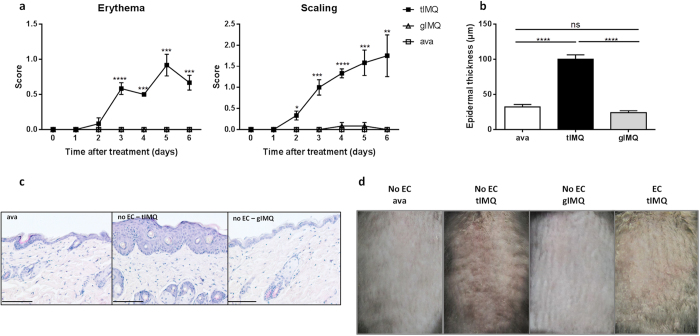
Oral gavage with Aldara did not restore the skin phenotype in mice wearing Elizabethan collars. Mice were treated with topical or gavaged Aldara or control vehicle (ava), and scored for psoriatic lesions as previously described^1^ (score 0–4). One group wore ECs, preventing oral uptake of IMQ. (**a**) Erythema and scaling were scored as described previously (score 0–4). (**b**) Epidermal hyperplasia in skin sections of treated mice taken on day 6, quantified with Volocity. (**c**) Representative skin sections of treated mice taken on day 6 stained with H&E. Scale bars represent 100 μm. (**d**) Skin rash of the back of mice after 6 days of treatment. Bars represent mean ± SEM; n = 6 per group. *EC: Elizabethan collar; tIMQ: topical IMQ; gIMQ: gavaged IMQ; white bar/open square curve = topical ava; black bar/curve = tIMQ; grey bar/open rectangle curve = gIMQ.*

**Figure 5:**
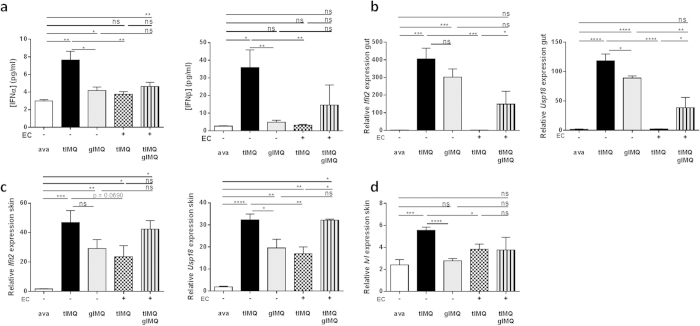
Gavaged IMQ results in a cutaneous and intestinal interferon signature. Mice with or without Elizabethan collars (ECs) were treated with either topical or oral Aldara, or a combination hereof. (**a**) Using Luminex technology, IFNα (left) and IFNβ (right) were measured in serum 4 hours after treatment. (**b**) Relative expression of *Ifit2* and *Usp18* RNA 4 hours after treatment in the gut. (**c**) Relative expression of *Ifit2* and *Usp18* in whole skin lysates 4 hours after treatment. (**d**) Relative expression of psoriatic markers genes Involucrin (*Ivl*) in whole skin lysates 4 hours after treatment. Bars represent mean ± SEM and qPCR data are normalized to stable reference genes; n = 5–6 per group. *EC: Elizabethan collar; tIMQ: topical IMQ; gIMQ: gavaged IMQ; white bar = topical ava; black bar = without EC, tIMQ; grey bar = without EC, gIMQ; dotted bar = with EC, tIMQ; striped bar = with EC, tIMQ and gIMQ*.

These observations provoked the question whether oral administration of Aldara is sufficient to affect the skin’s integrity and inflammatory status. Therefore, we compared topical application of Aldara to gavaging of Aldara on erythema, scaling and epidermal hyperplasia. Yet, mice gavaged with Aldara developed no signs of skin lesions, in contrast to mice treated topically with Aldara (Fig. 4a–c). Figure 4d displays the lesions on the back skin after 6 days, with no detectable lesions when Aldara is administered orally. As a control group we included a group of mice treated topically with the vehicle cream (ava), where, similarly to the gavaged mice, no skin lesions developed since a previous paper reported that the base cream of Aldara could induce skin inflammation itself^8^. Type I IFNs were not induced in circulation when Aldara was gavaged without topical application, whereas combination with topical treatment increased both IFNα and IFNβ levels in serum (Fig. 5a). At the molecular level, we investigated the transcriptional upregulation of *Ifit2* and *Usp18* in the gut and skin. In the intestinal system, mice without collars displayed increased mRNA levels of both genes in response to topical and gavaged Aldara, separately (Fig. 5b). No induction was detected in mice treated with the vehicle cream (ava). The increase in gavaged mice was less than the topical group, which reached statistical significance for *Usp18*. Mice wearing ECs did not display any increase of *Ifit2* or *Usp18*, whereas mice receiving concomitant topical and oral Aldara showed insignificant upregulation (Fig. 5b, dotted and striped bar). Cutaneous upregulation of *Ifit2* and *Usp18* was significant in response to topical and oral Aldara, separately, though less in the gavaged mice (Fig. 5c). When ECs were applied, Ifit2 and Usp18 were induced as well during topical treatment, though to a lesser extent than when mice were simultaneously gavaged (Fig. 5c, dotted and striped bar). The effect of gavaged Aldara on *Ivl* expression was not detectable, even in combination with topical treatment (Fig. 5d). Still, these data reflect the need for both cutaneous and intestinal triggering to have full-blown interferon activity in the skin.

In conclusion, topical treatment with Aldara induces a psoriasiform skin inflammation, but the mode of action partially depends on ingestion of the chemical, which leads to a systemic response. Preventing oral uptake blocks not only the systemic effects, but also affects the local cutaneous inflammation, indicating that the systemic response must be considered as an amplification loop. Moreover, these results raise concern about other products applied topically in mouse models if mice can ingest the product as well, such as TPA and acetone, and how the gut affects the skin phenotype. The relevance of this model and possibly other skin disease models employing topical chemical treatment should therefore be reconsidered.

## Methods

### Mice

C57BL6/J mice were purchased from Janvier (Le Genest-St Isle, France). Albino (tyr^c2J^) C57BL/6 IFNβ-reporter mice (ifnb1^tm2.2lien^), as well as control Albino (tyr^c2J^) C57BL/6 have been previously described^7^. Briefly they have a luciferase gene targeted to the *IFN-β* locus to visualize IFNβ expression. All animals were maintained in an SPF temperature-controlled, air-conditioned animal house with a 14- to 10-hour-light/dark cycle and received food and water *ad libitum*. Experiments were performed in the SPF animal house on 8–12 week old mice. All animal experiments were approved by the institutional ethics committee for animal welfare of the Faculty of Sciences, Ghent University, Belgium or the Niedersächsisches Landesamt für Verbraucherschutz und Lebensmittelsicherheit (LAVES), Germany and followed the European guidelines.

### Elizabethan collars

Elizabethan collars (ECs) were purchased from Harvard Apparatus (France). Mice were briefly sedated with isoflurane and the ECs were applied before Aldara cream was topically applied on their back or after the mice were orally gavaged with Aldara cream.

### Aldara-induced psoriasis-like skin lesions and scoring of symptoms

Mice were briefly sedated with isoflurane and the back skin of mice was shaved. Two days later, a dose of 62,5 mg of Aldara cream (5% IMQ, 3 M Pharmaceuticals) was applied daily on the back for 7 consecutive days. In order to investigate the effect of orally administered Aldara cream, mice were daily orally gavaged with 10 mg/ml Aldara cream, dissolved in 200 μl PBS. Mice were scored daily based on a modified PASI score system as described previously^1^. In brief, to describe the severity of psoriasis, erythema (redness of the skin) and scaling were scored “blindly” on a scale from 0 to 4: 0, none; 1, slight; 2: moderate; 3: marked; 4: very marked.

### Optical imaging

Mice were i.v. injected with 150 mg/kg D-luciferin in PBS, anesthetized with isoflurane and optical imaging was performed 4 h after Aldara treatment with an IVIS 200 System (Perkin Elmer). After *in vivo* imaging the mice were sacrificed and the intestine was isolated and imaged individually. Data were analyzed with Living Image 4.4 (Perkin Elmer) and are presented with the respective scales in p/sec/cm^2^/sr.

### Splenomegaly

Mice that had been treated daily with Aldara cream for seven consecutive days were weighted and killed. Their spleen was isolated and weighted separately. Splenomegaly was assessed by calculating the ratio of the weight of the spleen and bodyweight.

### Detection of IFN

Mice were treated with Aldara and euthanized for blood, small intestine, spleen and skin collection at the indicated times. Blood was collected through cardiac puncture and allowed to clot at 4 °C overnight. Serum was collected after removal of the clot and centrifugation and stored at −80 °C until assayed. Luminex technology was used to detect IFNα and IFNβ in serum (Mouse IFN alpha/beta Platinum (Procarta)). All above-mentioned techniques were performed according to the manufacturers’ instructions.

### RT-qPCR

Skin, small intestine and spleen samples were collected in RNA Later (Ambion). RNA from small intestine and spleen was isolated with the RNeasy Mini Kit (Qiagen) according to the manufacturer’s instructions. RNA from skin was isolated with the RNeasy Mini Kit (Qiagen) according to the manufacturer’s instructions. RNA concentration was measured with the Nanodrop1000 (Thermo Scientific). Quantitative real-time PCR (qPCR) was performed using SensiFast^TM^ Sybr NO-ROX Kit (Bioline) and the LightCycler 480 (Roche). Expression levels were normalized to the expression of the two most stable reference genes, which were determined for each condition using geNorm (qBase, Biogazelle). Reference genes were 1) spleen: Glyceraldehyde 3-phosphate dehydrogenase (*Gapdh*) and Ubiquitin (*Ubc*) 2) skin: Hypoxanthine-guanine phosphoribosyltransferase (*Hprt*) and ribosomal protein (*Rpl*) 3) small intestine: *Hprt* and *Rpl*. Values are represented as relative expression normalized to the geometric mean of the two selected most stable reference genes.

### Primer sequences and references genes

Primers used were *Mx1* (Fw 5′-GACCATAGGGGTCTTGACCAA-3′, Rev 5′-AGA CTTGCTCTTTCTGAAAAGCC-3′), *Usp18* (Fw 5′ AGCCCTCATGGTCTGGTTGGTT-3′, Rev 5′-GCACTCCGAGGCACTGTTATCC-3′), *Ifit2* (Fw 5′-AAATGTCATGG GTACTGGAGTT-3′, Rev 5′-ATGGCAATTATCAAGTTTGTGG-3′), *Tnf* (Fw 5′-ACCCTGGTATGAGCCCATATAC-3′, Rev 5′-ACACCCATTCCCTTCACAGAG-3′), *Ifnb* (Fw 5′-GCACTGG GTGGAATGAGACT-3′, Rev 5′-AGTGGAGAGCAGTTGAGGACA-3′), *Ifna* (Fw 5′-TCTGATGCAGCAGGTGGG-3′, Rev 5′-AGGGCTCTCCA GACTTCTGCTCTG-3′), *Ivl* (Fw 5′-ATGTCCCATCAACACACACTG-3′, Rev 5′-TGGAG TTGGTTGCTTTGCTTG-3′), *Gapdh* (Fw 5′-TGAAGCAGGCATCTGAGGG-3′, Rev 5′-CGAAGGTGGAAG AGTGGGAG-3′), *Ubc* (Fw 5′-AGGTCAAACAGGAAGACAGAC GTA-3′, Rev 5′-TCACACCCAAGAACAAGCACA-3′), *Hprt* (Fw 5′-AGTGTTGGATACAGG CCAGAC-3′, Rev 5′-CGTGATTCAAATCCCTGAAGT-3′) and *Rpl* (Fw 5′ CCTGCTGCT CTCAAGGTT-3′, Rev 5′ TGGTTGTCACTGCCTCGTACTT-3′).

### Statistical analysis

Results (mean ± SEM) were compared with a two-tailed Student’s t-test in most cases unless indicated differently. P values < 0.05 were considered significant. *0.01 < p < 0.05; **0.001 < p < 0.01; ***0.0001 < p < 0.001, ****p ≤ 0.0001.

## Additional Information

**How to cite this article**: Grine, L. *et al.* Topical imiquimod yields systemic effects due to unintended oral uptake. *Sci. Rep.* **6**, 20134; doi: 10.1038/srep20134 (2016).
